# Supplementary material for: Splice-Junction-Based Mapping of Alternative Isoforms in the Human Proteome
Source: Cell Rep. Author manuscript; Available in PMC 2020 Jan 15. (PMC6961840; doi:10.1016/j.celrep.2019.11.026)
Supplement: 2 [file NIHMS1546469-supplement-2.zip › DF1/PXD006675/RightAtrium/RightAtrium_35_TTN_ETPPVEVPGGEK.pdf]

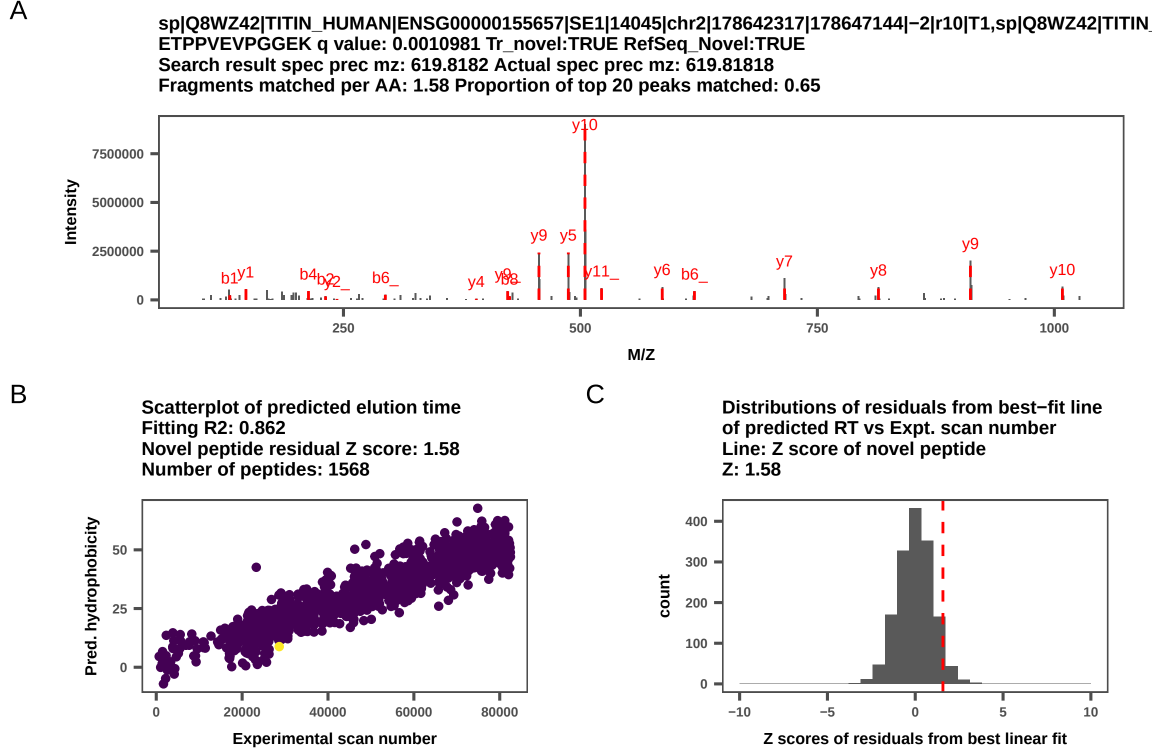
A
sp|Q8WZ42|TITIN_HUMAN|ENSG00000155657|SE1|14045|chr2|178642317|178647144|−2|r10|T1,sp|Q8WZ42|TITIN_
ETPPVEVPGGEK q value: 0.0010981 Tr_novel:TRUE RefSeq_Novel:TRUE
Search result spec prec mz: 619.8182 Actual spec prec mz: 619.81818
Fragments matched per AA: 1.58 Proportion of top 20 peaks matched: 0.65
Intensity
7500000
5000000
2500000
0
M/Z
250
500
750
1000
y10
y9
y5
y9
y7
y8
y10
y6
b6_
y11_
b1
y1
b4
b2
y2_
b6_
y4
B
Scatterplot of predicted elution time
Fitting R2: 0.862
Novel peptide residual Z score: 1.58
Number of peptides: 1568
Pred. hydrophobicity
50
25
0
Experimental scan number
0
20000
40000
60000
80000
C
Distributions of residuals from best−fit line
of predicted RT vs Expt. scan number
Line: Z score of novel peptide
Z: 1.58
count
400
300
200
100
0
Z scores of residuals from best linear fit
−10
−5
0
5
10
